# Altered Neuronal Activity in the Central Nucleus of the Amygdala Induced by Restraint Water-Immersion Stress in Rats

**DOI:** 10.1007/s12264-018-0282-y

**Published:** 2018-08-31

**Authors:** Feng He, Hongbin Ai, Min Wang, Xiusong Wang, Xiwen Geng

**Affiliations:** 1 Shandong Provincial Key Laboratory of Animal Resistance Biology, College of Life Sciences, Shandong Normal University, Jinan, 250014 China; 2 Advanced Materials Genome Innovation Team, Advanced Materials Institute, Qilu University of Technology (Shandong Academy of Sciences), Jinan, 250353 China

**Keywords:** Central nucleus of the amygdala, Restraint water-immersion stress, Neuronal activity, Corticotropin-releasing hormone, Gastric ulceration

## Abstract

Restraint water-immersion stress (RWIS), a compound stress model, has been widely used to induce acute gastric ulceration in rats. A wealth of evidence suggests that the central nucleus of the amygdala (CEA) is a focal region for mediating the biological response to stress. Different stressors induce distinct alterations of neuronal activity in the CEA; however, few studies have reported the characteristics of CEA neuronal activity induced by RWIS. Therefore, we explored this issue using immunohistochemistry and *in vivo* extracellular single-unit recording. Our results showed that RWIS and restraint stress (RS) differentially changed the c-Fos expression and firing properties of neurons in the medial CEA. In addition, RWIS, but not RS, induced the activation of corticotropin-releasing hormone neurons in the CEA. These findings suggested that specific neuronal activation in the CEA is involved in the formation of RWIS-induced gastric ulcers. This study also provides a possible theoretical explanation for the different gastric dysfunctions induced by different stressors.

## Introduction

Restraint water-immersion stress (RWIS) is widely accepted as a useful procedure to induce gastric ulceration in rats [1–3]. Based on studies of the brainstem circuits regulating gastric function [4–6], altered neuronal activity in the dorsal vagal complex (DVC) is considered to be responsible for the stress-induced gastric acid hypersecretion and gastric hypercontractility, which are essential factors for the formation of stress-induced gastric mucosal damage [7]. Anatomical studies have demonstrated that both the nucleus of the solitary tract (NST) and the dorsal motor nucleus of the vagus (DMV), involved in the regulation of gastrointestinal activity, receive afferents from the central nucleus of the amygdala (CEA) [8, 9]. Physiological studies have demonstrated that electrical stimulation of different regions of the CEA induces increased gastric acid secretion [10], increased or decreased gastric motility [11, 12], gastric ulceration [13], and altered activity of neurons in the NST and DMV [9, 12]. Together, both anatomical and physiological studies suggest that the CEA plays an important role in the mechanisms underlying the formation of stress-induced ulcers.

The effects of different stressors on neuronal activity in the CEA are different. As demonstrated previously [14–17], stressors such as immobilization, starvation, pain, hypoglycemia, and foot shock induce c-Fos expression at different levels in the CEA, but stressors such as cold and blood-loss do not. However, it is unclear whether RWIS changes neuronal activity in the CEA.

c-Fos immunohistochemistry has been used as a functional anatomical mapping tool to identify cells and serves as a marker of neuronal activation [18, 19]. Neuronal activation can also be evaluated by the firing rate and firing pattern in extracellular recordings of neuronal spikes [20, 21]. The firing rate, defined as the temporal average number of spikes, is used to evaluate neuronal activity and the firing pattern described by the coefficient of variance (CV) is used to evaluate neuronal discharge regularity. Therefore, we used c-Fos immunohistochemistry and extracellular spike recording to assess the alterations of neuronal activity.

We hypothesized that specific and distinct neuronal activation in the CEA is involved in the formation of RWIS-induced gastric ulcers.

## Materials and Methods

### Animals

A total of 75 male Wistar rats weighing 250 g–300 g were used in experiments. The rats were purchased from the Experimental Animal Center of Shandong University and kept in a temperature-controlled room (22 ± 2°C) under a natural day/night cycle. All the animals were housed in groups of 3–4 per cage and supplied with food and water *ad libitum* for one week before experiments began. All experimental procedures were conducted in accordance with the Guide for the Care and Use of Laboratory Animals (NIH Guidelines) and were approved by the Animal Experimental Ethics Committee of Shandong Normal University. All efforts were made to minimize the number and suffering of the experimental animals.

### Stress Protocols

The rats were randomly divided into three groups: RWIS, RS, and controls. All rats were fasted for 24 h before the start of an experiment. RWIS was carried out as previously described [22]. Briefly, after light anesthesia with ethyl ether, the four limbs of each rat were bound gently and securely to a wooden board. When the rats regained consciousness, they were immersed vertically in water (21 ± 1°C) to the level of the xiphoid for 3 h. In the RS group, except for immersion in water, each rat was treated similarly to those in the RWIS group. The rats in the control group were free from RS or RWIS stress.

### Electrophysiological Recording and Analysis

After the stress procedure, the rats were anesthetized with 20% urethane (1.2 g/kg, i.p.). Local anesthesia with 2% xylocaine was used before scalp incision. The head was positioned in a stereotactic frame (68002, RWD Life Science, Shenzhen, China) with bregma and lambda adjusted to the same horizontal plane. A heating blanket controlled by an anal probe prevented hypothermia during surgery.

A limited craniotomy was made where the recording microelectrode was to be implanted. Neuronal spikes were recorded extracellularly with single-barrel glass microelectrodes (tip diameter 1 μm–2 μm; resistance 8 MΩ–15 MΩ) filled with 0.5 mol/L sodium acetate and 2% pontamine sky blue (PSB). After the dura was punctured with a needle, the microelectrode was implanted into the lateral CEA (CEl) using a microdrive (68105, RWD Life Science, China) at 10 μm/s (coordinates: 2.0 mm–2.8 mm posterior to bregma, 4.3 mm–4.8 mm lateral to the midline, and 7.0 mm–7.6 mm ventral to the dura mater) or the medial CEA (CEm, coordinates: 1.8 mm–2.5 mm posterior to bregma, 3.5 mm–4.0 mm lateral to the midline, and 7.2 mm–7.7 mm ventral to the dura mater) [23]. The brain surface was covered with 3% agar in saline to reduce the influence of ventilation and heartbeats.

The signals were amplified with a microelectrode bridge amplifier (ME200A, Chengdu Taimeng, China), recorded at a sampling rate of 10 kHz, and band-pass filtered at 160 Hz–5000 Hz by a Biological Experimental System (BL-420F, Chengdu Taimeng, China). When a neuron was encountered, signals were recorded for 5 min. Only stable recordings with signal-to-noise ratio > 2.0 were stored and sorted using Offline Sorter (Plexon, Inc., Dallas, TX), based on principal component analysis and the unit waveform and duration. Saved spike signals were then exported to NeuroExplorer 4 (NEX Technologies,Winston-Salem, NC) for inter-spike interval (ISI) analysis, from which we obtained the mean firing rate (spikes/s) and CV. The CV was calculated as the standard deviation of the ISIs divided by the mean ISI to describe the degree of regularity of neuronal discharge. CV values close to 0 indicate regular firing patterns, while CV values close to or > 1 indicate irregular firing patterns [24].

### Histology

**Fig. 1:**
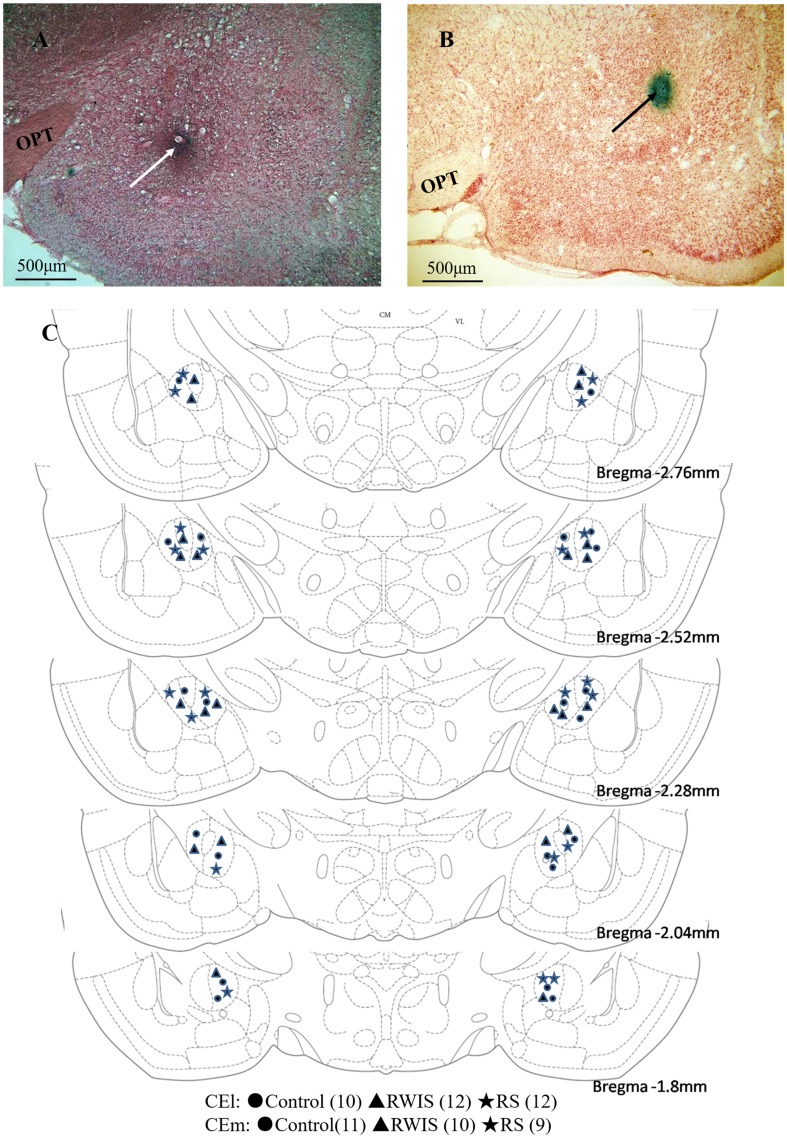
Histological verification of recording sites. **A**–**B** Two representative examples of coronal sections stained with neutral red, photographed at low magnification, demonstrating the positions of microelectrode tips in the medial part of the central nucleus of the amygdala (white arrow) and the lateral part of the central nucleus of the amygdala (black arrow). **C** Schematic reconstructions of recording locations in the control (circles), RWIS (triangle), and RS groups (stars). Distances of coronal sections posterior to bregma are according to the rat brain atlas of Paxinos and Watson [23]. OPT, optic tract.

The positions of microelectrode tips were verified histologically after the electrophysiological recordings were finished. The procedure followed that described in our previous study [12]. Briefly, anodic direct current (0.01 mA, 20 min) was passed through the recording electrode to form an iron deposit of phosphate buffered saline (PBS) in the recording site. Then, the rats were euthanized with an overdose of urethane and perfused transcardially with 150 mL of 0.9% NaCl and 250 mL of 4% paraformaldehyde followed by removal of the brain. After post-fixing in a mixture of 4% paraformaldehyde and 20% sucrose for > 24 h, the brains were processed for frozen coronal sectioning (30 μm) and the sections were stained with neutral red. Only data collected from electrodes in the correct positions were used for further statistical analysis. The tip sites in the CEA (CEm and CEl) of all rats used for further analysis are shown in Fig. 1.

### Immunohistochemistry

An immunohistochemical test of c-Fos and a dual immunohistochemical test of c-Fos and corticotropin-releasing hormone (CRH) were performed in brain slices from 10 rats in each group. After the stress procedure, rats were anesthetized with an overdose of urethane and then transcardially perfused with 200 mL of 0.01 mol/L PBS (pH 7.4) followed by 500 mL freshly-prepared 4% paraformaldehyde in 0.1 mol/L phosphate buffer (4°C). After perfusion, the brains were removed and post-fixed in 4% paraformaldehyde for 3 h, and cryoprotected overnight in 20% sucrose in 0.01 mol/L PBS at 4°C. Coronal serial sections were cut at 30 μm on a cryostat and stored in PBS for immunohistochemical tests.

c-Fos immunoreactivity (IR) was determined as previously reported [2]. Briefly, coronal sections containing the CEA were washed and incubated in PBS containing 0.5% Triton-X100 and 5% normal goat serum at room temperature for 30 min, followed by primary rabbit anti-c-Fos antibody (sc-52, 1:1000, Santa Cruz Biotechnology Inc., Dallas, TX) for 24 h at 4°C. Subsequently, the sections were incubated with biotinylated goat anti-rabbit IgG (Zymed Laboratories, San Francisco, CA) for 1 h and then with streptavidin-biotin-horseradish peroxidase complex (Zymed Laboratories) for 1 h, both at room temperature. Then the sections were immersed in diaminobenzidine hydrochloride (DAB, Sigma Chemical Co., St. Louis, MO) for 2–4 min, yielding a brown reaction product in the nucleus. Lastly, the stained sections were mounted on gelatin-coated glass slides, air-dried overnight, dehydrated with 75%–100% alcohols, cleared in xylene, and coverslipped with Permount mounting medium for microscopic observation.

c-Fos-IR of CRH neurons was determined using a dual immunohistochemical method. First, sections were stained with c-Fos-IR according to the above procedure, but an intensifying DAB solution containing 0.05% cobalt chloride and 0.05% nickel ammonium sulfate was applied instead, yielding a dark reaction product in the nucleus. Second, these c-Fos-IR sections were immunohistochemically reacted with rabbit anti-CRH (ab11133, 1:1000, Abcam plc, Cambridge, UK). All the steps were the same except for the different antibody. The CRH-immunoreactive products were visualized by reaction with normal DAB solution, yielding a brown reaction product in the cytoplasm.

Selected photomicrographs were captured on a BX51 Olympus microscope coupled to an DP70 camera (Olympus Corp., Tokyo, Japan). We used the nuclear boundaries defined in the rat brain stereotaxic atlas of Paxinos and Watson [23]. Image-Pro Plus 6.0 (Media Cybernetics Inc., Rockville, MD) was used to count c-Fos-IR neurons and c-Fos + CRH-IR neurons in the CEl and CEm. The number of immunoreactive neurons was counted from three non-consecutive sections per animal and the average number in 0.01 mm^2^ was reported as the immunohistochemical measure.

### Evaluation of Gastric Mucosal Lesions

The stomachs from 10 rats in each group were removed, incised along the greater curvature, and then rinsed with normal saline. Gastric lesions were identified with a magnifying lens and evaluated as the erosion index (EI) according to the methods of Guth [25]. Briefly, EI scores were given according to the length of the lesion: ≤ 1 mm was scored as 1, 1 mm to ≤ 2 mm as 2, and so on. The score was multiplied by 2 when the width of the lesion was > 1 mm. The cumulative scores of all lesions in a rat served as the EI of that rat.

### Statistical Analysis

The data are presented as mean ± SEM and analyzed with SPSS 18.0 statistical software (SPSS Inc., Chicago, IL). We used one-way analysis of variance (ANOVA) followed by the Bonferroni method (equal variance assumed) or Tamhane’s T2 test (equal variance not assumed). *P*-values < 0.05 were considered to be statistically significant.

## Results

### c-Fos Expression of CEA Neurons

**Fig. 2:**
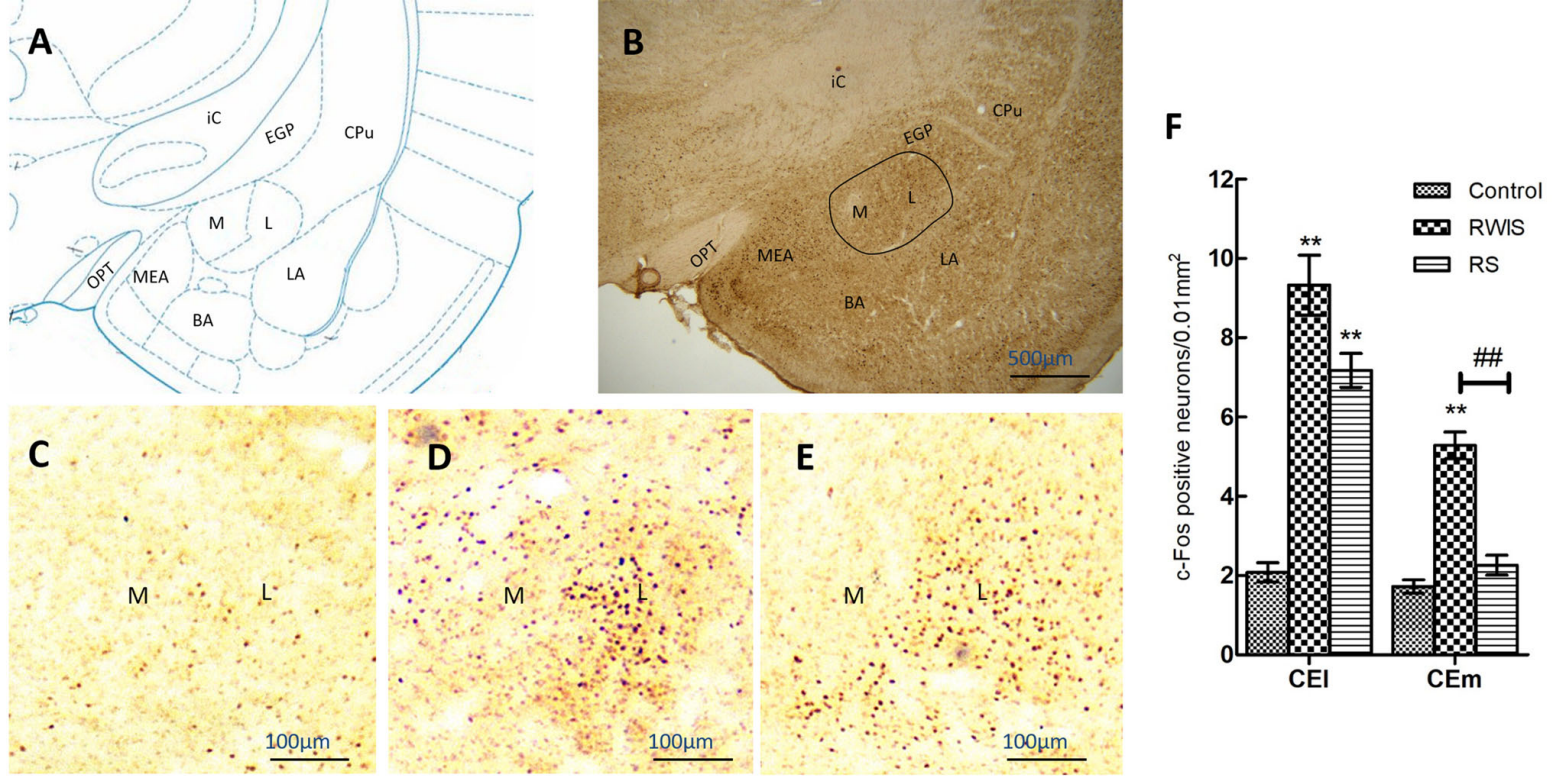
Expression of c-Fos in the central nucleus of the amygdala (CEA). **A** Reference plane (2.28 mm posterior to bregma) for the CEA. **B** Example image of c-Fos expression in the CEA (outlined) and the adjacent tissue from the RWIS group at 40× magnification. **C**–**E** Example images of c-Fos expression in the CEA from the control (**C**), RWIS (**D**), and RS groups **(E)** at 100× magnification. **F** Comparisons of the c-Fos expression between groups. Data represent mean ± SEM (*n* = 10/group). **P < 0.01 compared with control; ^##^*P* < 0.01 between RWIS and RS groups. M, medial CEA (CEm); L, lateral CEA (CEl); MEA, medial amygdala; BMA, basomedial amygdala; BLA, basolateral amygdala; OPT, optic tract; ic, internal capsule; EGP, external globus pallidus; CPu, caudate putamen.

The results of c-Fos expression of neurons in the CEA (CEl and CEm) from each group (*n* = 10/group) are shown in Fig. 2. Example images of c-Fos expression in the CEA of the control (Fig. 2C), RWIS (Fig. 2D), and RS groups (Fig. 2E) were taken from brain sections 2.28 mm posterior to bregma according to the reference plane (Fig. 2A, B). One-way ANOVA showed significant differences in the number of c-Fos-positive neurons between the three groups in both the CEl (*F*(2,27) = 50.11, *P* < 0.001) and the CEm (*F*(2,27) = 53.10, *P* < 0.001). Compared with control treatment, both RWIS and RS induced significant increases in c-Fos expression in the CEl (9.32 ± 0.76 and 7.17 ± 0.43 *vs* 2.08 ± 0.24, both *P* < 0.001), but only RWIS did so in the CEm (5.28 ± 0.34 *vs* 1.72 ± 0.17, *P* < 0.001). Furthermore, compared with RS, RWIS induced significant increases in c-Fos expression in the CEm (5.28 ± 0.34 *vs* 2.26 ± 0.25, *P* < 0.001), but not in the CEl (9.32 ± 0.76 *vs* 7.17 ± 0.43, *P* = 0.081) (Fig. 2C–F).

### Firing Rate and CV of CEA Neurons

**Fig. 3:**
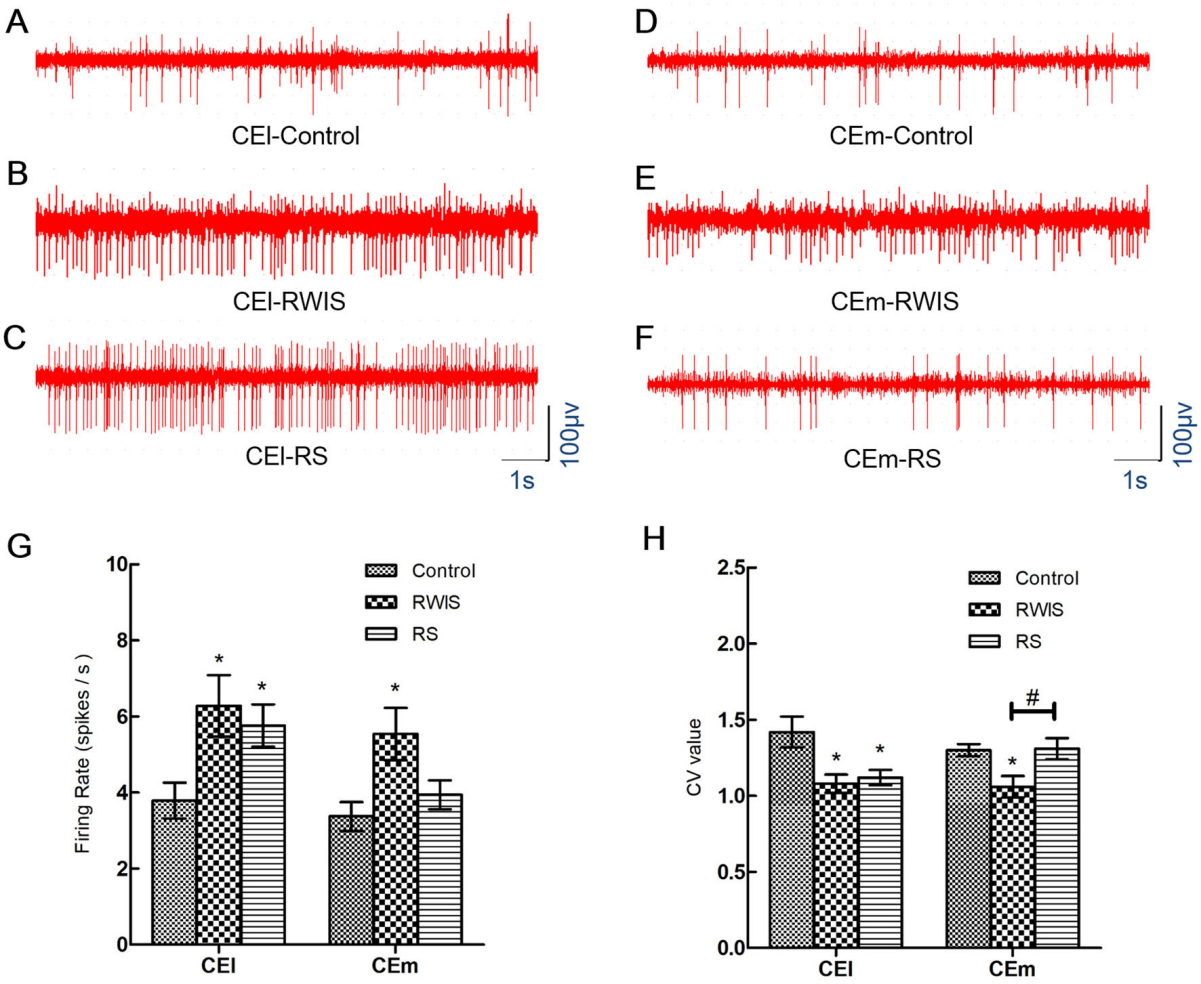
Firing rates and CVs of neuronal spikes in the CEA. **A**–**F** Representative original recordings of spikes from CEl and CEm neurons in the control, RS, and RWIS groups. **G**–**H** Comparisons of the firing rates and CVs between groups. Data represent the mean ± SEM. *P < 0.05 compared with control; ^#^*P* < 0.05 between RWIS and RS groups.

After histological analysis and spike-sorting, a total of 118 neurons recorded from the CEl and 115 neurons recorded from the CEm were used in the statistical analysis. There were 36 neurons from 10 control rats, 40 from 12 RWIS rats, and 42 from 12 RS rats for CEl recording; and 38 neurons from 11 control rats, 40 from 10 RWIS rats, and 37 from 9 RS rats for CEm recording. Representative original traces of neuronal spikes are shown in Fig. 3A–F. Comparisons of the firing rate and CV value of the CEl/CEm neurons in each group are shown in Fig. 3G, H. One-way ANOVA showed significant differences in firing rate both in the CEl (*F*(2,115) = 4.019, *P* = 0.032) and CEm (*F*(2,112) = 4.865, *P* = 0.009) among the three groups (Fig. 3G). The firing rate of CEl neurons in RWIS and RS rats were significantly higher than that in control rats (6.28 ± 0.81 and 5.76 ± 0.56 *vs* 3.79 ± 0.47, *P* = 0.03 and *P* = 0.027, respectively). But there was no significant difference between RWIS and RS rats (*P* = 0.933). Meanwhile, the firing rate of CEm neurons in RWIS rats was significantly higher than that in control rats (5.54 ± 0.69 *vs* 3.37 ± 0.38, *P* = 0.023), but there was no significant difference between RS and control rats (3.94 ± 0.38 *vs* 3.37 ± 0.38, *P* = 0.649). One-way ANOVA showed significant differences in the CV both in the CEl (*F*(2,115) = 6.182, *P* = 0.003) and CEm (*F*(2,112) = 5.313, *P* = 0.006) among the three groups (Fig. 3H). The CVs of CEl neurons in RWIS and RS rats were significantly lower than that in control rats (1.08 ± 0.06 and 1.12 ± 0.05 *vs* 1.42 ± 0.10, *P* = 0.019 and *P* = 0.037, respectively), but there was no significant difference between RWIS and RS rats (*P* = 0.946). Meanwhile, the CV of CEm neurons in RWIS rats was significantly lower than that in control or RS rats (1.06 ± 0.07 *vs* 1.30 ± 0.04 and 1.31 ± 0.07, *P* = 0.014 and *P* = 0.037, respectively). There was no significant difference between RS and control rats (1.31 ± 0.07 *vs* 1.30 ± 0.04, *P* = 0.998).

### c-Fos Expression of CRH Neurons in the CEA

**Fig. 4:**
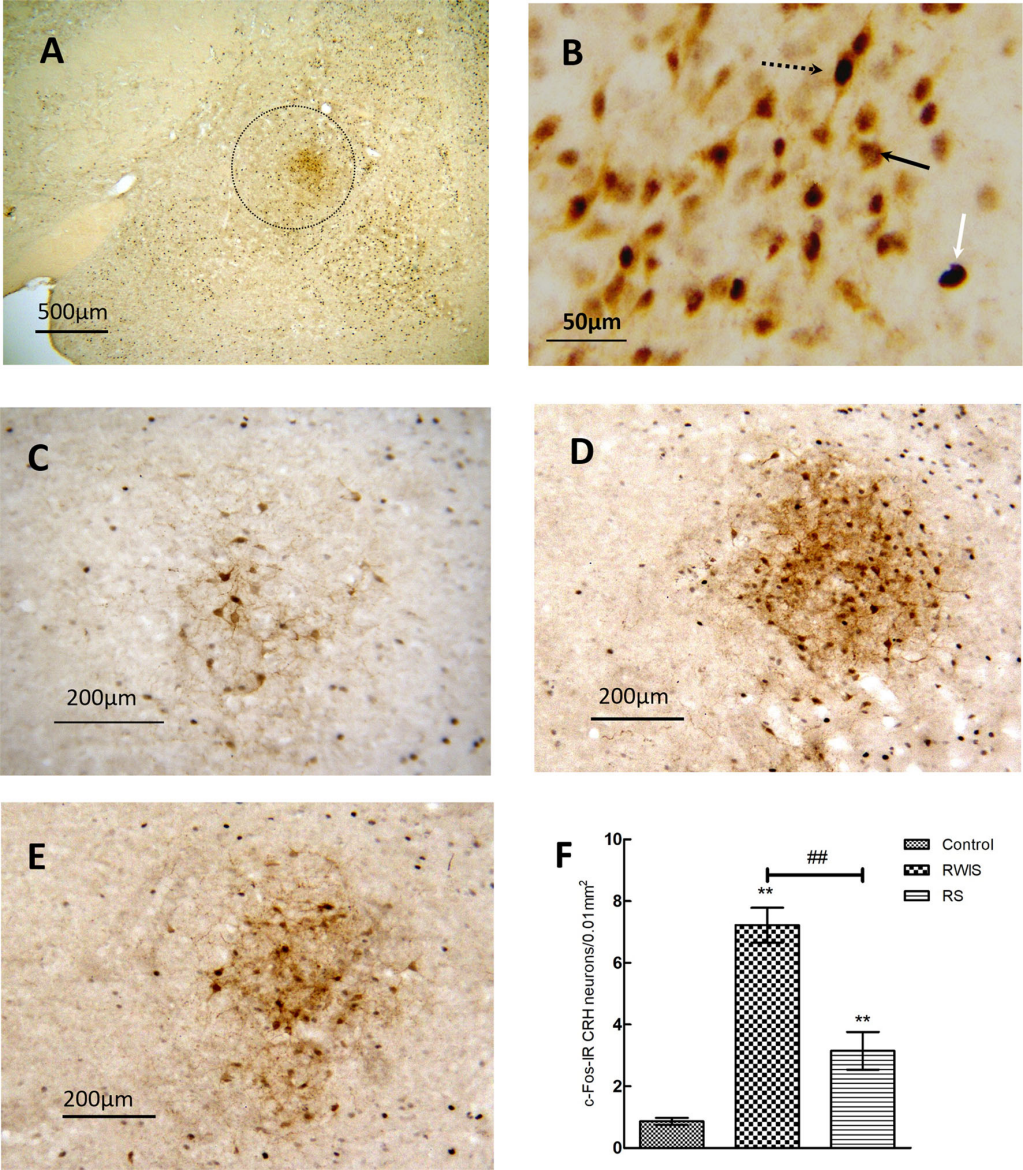
Expression of c-Fos in CRH neurons in the CEA. **A** Example image of c-Fos expression in CRH neurons in the CEA (outlined) from the RWIS group at 40× magnification. **B** Example image of dual immunohistochemical staining for c-Fos and CRH-IR neurons in the CEA. White arrow, a c-Fos-IR neuron; black arrow, a CRH-IR neuron; dotted black arrow, a c-Fos-IR CRH neuron. **C**–**E** Example images of c-Fos expression in CRH neurons from the CEA of control **(C)**, RWIS **(D),** and RS groups **(E)**, at 200× magnification. **F** Comparisons of number of c-Fos-IR CRH neurons between groups. Data represent the mean ± SEM (*n* = 10/group). **P < 0.01 compared with control; ^##^*P* < 0.01 between RWIS and RS groups.

The results for c-Fos expression in CRH neurons in the CEA from each group (*n* = 10/group) are shown in Fig. 4. CRH-immunoreactive neurons were mostly clustered in the CEl and rarely seen in the CEm (Fig. 4A), consistent with previous reports on the distribution of CRH neurons in the CEA [26, 27]. So, in the subsequent analysis of the c-Fos expression of CRH neurons, we used the entire CEA without medial/lateral distinction. Representative CRH and/or c-Fos-immunoreactive neurons are shown in Fig. 4B, together with example images of c-Fos expression in CRH neurons in the CEA from the control, RWIS, and RS groups (Fig. 4C–E). One-way ANOVA showed significant differences in the number of c-Fos-positive CRH neurons in the CEA (*F*(2,27) = 44.145, *P* < 0.0001) among the three groups (Fig. 4F). Compared with controls, both RWIS and RS induced significant increases in the number of c-Fos-positive CRH neurons (7.22 ± 0.56 and 3.15 ± 0.61 *vs* 0.86 ± 0.12, *P* < 0.001 and *P* = 0.013, respectively). Furthermore, compared with RS, RWIS induced a significant increase in the number of c-Fos-positive CRH neurons (7.22 ± 0.56 *vs* 3.15 ± 0.61, *P* < 0.001).

### Gastric Mucosal Damage

**Fig. 5:**
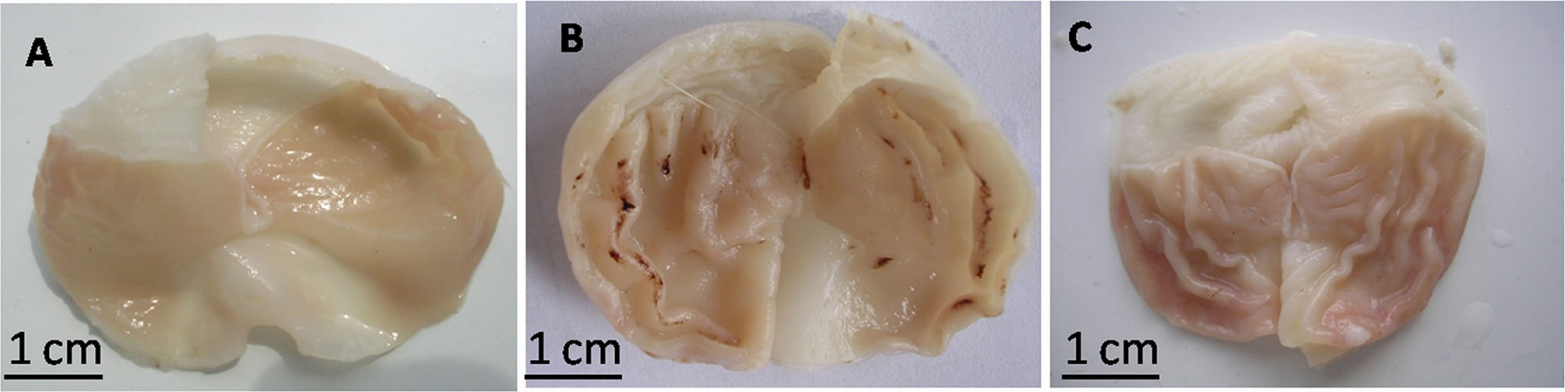
Representative gastric mucosae from control (**A**), RWIS (**B**), and RS (**C**) groups. Gastric mucosal damage was induced only in the RWIS group.

Representative gastric mucosae from each group (*n* = 10/group) are shown in Fig. 5A–C. There were no macroscopic gastric mucosal lesions in the control and RS groups. Scattered spots or linear hemorrhages and lesions were clearly visible in the corpus mucosa along the folds in the RWIS group and the mean EI was 20.90 ± 2.43.

## Discussion

The amygdala, an essential component of the limbic system, has received considerable attention due to its important role in stress-control circuitry [28, 29]. As the main output of the amygdala, the CEA is differentially activated by various stressors and deeply involved in autonomic regulation as well as stress-related behaviors [14, 30–32].

The possible role of the CEA in the formation of gastric ulcers has been a focus of previous reports. Electrical stimulation of the CEA induces vagus-dependent gastric ulceration, while bilateral lesions in the CEA reduce the severity of stress ulceration [13, 33]. Microinjection of neurotransmitters or neuropeptides including γ-aminobutyric acid, β-endorphin, dopamine, thyrotropin-releasing hormone, and CRH into the CEA results in the attenuation or aggravation of stress-induced gastric ulcers [34–37]. However, few reports have described the changes in neuronal activity in the CEA elicited by RWIS, a specific stress that has been demonstrated to be an acute procedure to induce gastric ulceration in rats in a few hours [38]. In the present study, our data confirmed that RWIS induced severe gastric mucosal damage in 3 h, while RS did not.

As far as the procedure is concerned, RWIS can be regarded as a compound stress model of RS and cold stress [38]. Previous studies have demonstrated that c-Fos expression in the CEA is increased by RS but not by cold stress [14, 17]. In the present study, both RWIS and RS induced strong c-Fos expression of CEl neurons. However, significant increase of c-Fos expression in CEm neurons was only induced by RWIS but not by RS. Considering that the output of CEA neurons terminating in the DVC is greater from the CEm than from the CEl [39–41], the differential changes of neuronal activity in the CEm and CEl could be worth further investigation to help explain the different gastric pathology induced by different stresses, such as RWIS and RS.

The specific response induced by RWIS in CEA neurons was also reflected in our analysis of spiking activity. Both RWIS and RS induced an increase in the mean firing rate of CEl neurons, but only RWIS induced an increase in the firing rate of CEm neurons, consistent with the changes in neuronal activity shown by c-Fos expression. As a marker of neuronal activity, c-Fos expression is often, but not always, associated with increased neuronal firing [42], so the electrophysiological data provided a more direct measurement of neuronal activity. A common means of evaluating the regularity of neuronal discharge is to calculate the CV values of the ISIs [24, 43]. The CVs of CEl and CEm neurons in each group were all > 1, revealing irregular firing patterns of CEA neurons in general. Furthermore, the CVs of CEl and CEm neurons in RWIS rats were lower than those in controls, suggesting less irregular firing patterns in RWIS rats. Alteration of the firing pattern induced by RWIS was similar to that induced by RS in the CEl; however, only RWIS induced significant alteration of the firing pattern in the CEm. Our previous study demonstrated that stimulation of the CEm significantly increases gastric motility [12], which is a major factor in the formation of stress ulcers. Together, these results suggest that the relationship between specific changes in CEA neuronal activity and RWIS-induced gastric pathology deserves further study.

In addition, we assessed the activity of CRH neurons in the CEA by dual immunostaining for c-Fos and CRH. CRH has been identified as a key neuropeptide responsible for initiating many of the endocrine, autonomic, and behavioral responses to stress [44, 45]. The CEA is a major extrahypothalamic source of CRH neurons [46], the activity of which is differently affected by different stresses. According to previous reports, stress-induced activation of CRH neurons occurs in acute RS [47] and chronic unpredictable mild stress [48], but not in acute audiogenic stress [49] and cold stress [50]. Our data showed that RWIS induced much more activation of CRH neurons in the CEA than RS, which has not been previously described. CRH neurons in the CEA project directly to several areas, including the hypothalamic paraventricular nucleus, the DVC, the bed nucleus of the stria terminalis, and the parabrachial nucleus, by which the CEA mediates the integration of abdominal and cutaneous pain, gastrointestinal motility, and anxiety [32, 51, 52]. Interestingly, our data suggested a possible correlation between stress-induced gastric ulceration and the activation of CEA CRH neurons. The exact role that the CEA CRH neurons play in stress-induced gastric ulceration deserves further investigation.

In summary, RWIS induced increases of neuronal activity and altered the firing pattern in both the medial and lateral CEA, as well as inducing the activation of CEA CRH neurons. These responses, especially in the CEm, induced by RWIS differed from those induced by RS, which could be involved in the formation of specific stress-induced pathology, such as gastric ulceration induced by RWIS. However, further studies are needed to explore the direct link between neuronal activity in the CEA and stress (i.e. RWIS)-induced gastric ulceration.

## References

[1] Sun H, Liu Z, Ma X (2016). Interactions between astrocytes and neurons in the brainstem involved in restraint water immersion stress-induced gastric mucosal damage. Neuroreport.

[2] Zhang YY, Zhu WX, Cao GH, Cui XY, Ai HB (2009). c-Fos expression in the supraoptic nucleus is the most intense during different durations of restraint water-immersion stress in the rat. J Physiol Sci.

[3] Sun HZ, Zheng S, Lu K, Hou FT, Bi JX, Liu XL (2017). Hydrogen sulfide attenuates gastric mucosal injury induced by restraint water-immersion stress via activation of KATP channel and NF-κB dependent pathway. World J Gastroentero.

[4] Travagli RA, Hermann GE, Browning KN, Rogers RC (2006). Brainstem circuits regulating gastric function. Annu Rev Physiol.

[5] Cruz MT, Murphy EC, Sahibzada N, Verbalis JG, Gillis RA (2007). A reevaluation of the effects of stimulation of the dorsal motor nucleus of the vagus on gastric motility in the rat. Am J Physiol-Reg.

[6] Zhou SY, Lu YX, Yao H, Owyang C (2008). Spatial organization of neurons in the dorsal motor nucleus of the vagus synapsing with intragastric cholinergic and nitric oxide/VIP neurons in the rat. Am J Physiol-Gastrl.

[7] Hayase M, Takeuchi K (1986). Gastric acid secretion and lesion formation in rats under water-immersion stress. Digest Dis Sci.

[8] Liubashina O, Jolkkonen E, Pitkänen A (2000). Projections from the central nucleus of the amygdala to the gastric related area of the dorsal vagal complex: a Phaseolus vulgaris-leucoagglutinin study in rat. Neurosci Lett.

[9] Zhang X, Cui J, Tan Z, Jiang C, Fogel R (2003). The central nucleus of the amygdala modulates gut-related neurons in the dorsal vagal complex in rats. J Physiol.

[10] Innes DL, Tansy MF (1980). Gastric mucosal ulceration associated with electrochemical stimulation of the limbic brain. Brain Res Bull.

[11] Liubashina O, Bagaev V, Khotiantsev S (2002). Amygdalofugal modulation of the vago-vagal gastric motor reflex in rat. Neurosci Lett.

[12] He F, Ai HB (2016). Effects of electrical stimulation at different locations in the central nucleus of amygdala on gastric motility and spike activity. Physiol Res.

[13] Henke PG, Ray A, Sullivan RM (1991). The amygdala. Digest Dis Sci.

[14] Pacak K, Palkovits M (2001). Stressor specificity of central neuroendocrine responses: implications for stress-related disorders. Endocr Rev.

[15] Pacak K, Palkovits M, Yadid G, Kvetnansky R, Kopin IJ, Goldstein DS (1998). Heterogeneous neurochemical responses to different stressors: a test of Selye’s doctrine of nonspecificity. Am J Physiol.

[16] Senba E, Ueyama T (1997). Stress-induced expression of immediate early genes in the brain and peripheral organs of the rat. Neurosci Res.

[17] Mohammad G, Chowdhury I, Fujioka T, Nakamura S (2000). Induction and adaptation of Fos expression in the rat brain by two types of acute restraint stress. Brain Res Bull.

[18] Furlong TM, McDowall LM, Horiuchi J, Polson JW, Dampney RA (2014). The effect of air puff stress on c-Fos expression in rat hypothalamus and brainstem: central circuitry mediating sympathoexcitation and baroreflex resetting. Eur J Neurosci.

[19] Gao HR, Zhuang QX, Zhang YX, Chen ZP, Li B, Zhang XY (2017). Orexin directly enhances the excitability of globus pallidus internus neurons in rat by co-activating OX1 and OX2 receptors. Neurosci Bull.

[20] Wang M, Qu Q, He T, Li M, Song Z, Chen F, Zhang X, Xie J, Geng X, Yang M (2016). Distinct temporal spike and local field potential activities in the thalamic parafascicular nucleus of parkinsonian rats during rest and limb movement. Neuroscience.

[21] Ma C, Ma X, Fan J, He J (2017). Neurons in primary motor cortex encode hand orientation in a reach-to-grasp task. Neurosci Bull.

[22] Zhao DQ, Ai HB (2011). Oxytocin and vasopressin involved in restraint water-immersion stress mediated by oxytocin receptor and vasopressin 1b receptor in rat brain. PLoS One.

[23] Paxinos G, Watson C (2005). The Rat Brain in Stereotaxic Coordinates.

[24] Geng X, Xie J, Wang X, Wang X, Zhang X, Hou Y (2016). Altered neuronal activity in the pedunculopontine nucleus: An electrophysiological study in a rat model of parkinson’s disease. Behav Brain Res.

[25] Guth PH (1992). Current concepts in gastric microcirculatory pathophysiology. Yale J boil Med.

[26] Pitts MW, Todorovic C, Blank T, Takahashi LK (2009). The central nucleus of the amygdala and corticotropin-releasing factor: insights into contextual fear memory. J Neurosci.

[27] Marchant NJ, Densmore VS, Osborne PB (2007). Coexpression of prodynorphin and corticotrophin-releasing hormone in the rat central amygdala: Evidence of two distinct endogenous opioid systems in the lateral division. J Comp Neurol.

[28] Ulrich-Lai YM, Herman JP (2009). Neural regulation of endocrine and autonomic stress responses. Nat Rev Neurosci.

[29] Kim MJ, Loucks RA, Palmer AL, Brown AC, Solomon KM, Marchante AN (2011). The structural and functional connectivity of the amygdala: from normal emotion to pathological anxiety. Behav Brain Res.

[30] Roozendaal B, McEwen BS, Chattarji S (2009). Stress, memory and the amygdala. Nat Rev Neurosci.

[31] Myers B, McKlveen JM, Herman JP (2012). Neural regulation of the stress response: the many faces of feedback. Cell Mol Neurobiol.

[32] Myers B, Greenwood-Van Meerveld B (2009). Role of anxiety in the pathophysiology of irritable bowel syndrome: importance of the amygdala. Front Neurosci.

[33] Henke PG (1988). Recent studies of the central nucleus of the amygdala and stress ulcers. Neurosci Biobehav Rev.

[34] Sullivan R, Henke P, Ray A, Hebert M, Trimper J (1989). The GABA/benzodiazepine receptor complex in the central amygdalar nucleus and stress ulcers in rats. Behav Neural Biol.

[35] Ray A, Henke PG (1990). Enkephalin-dopamine interactions in the central amygdalar nucleus during gastric stress ulcer formation in rats. Behav Brain Res.

[36] Mayer EA (2000). The neurobiology of stress and gastrointestinal disease. Gut.

[37] Henke PG, Sullivan RM, Ray A (1988). Interactions of thyrotropin-releasing hormone (TRH) with neurotensin and dopamine in the central nucleus of the amygdala during stress ulcer formation in rats. Neurosci Lett.

[38] Landeira-Fernandez J (2004). Analysis of the cold-water restraint procedure in gastric ulceration and body temperature. Physiol Behav.

[39] Ehrlich I, Humeau Y, Grenier F, Ciocchi S, Herry C, Lüthi A (2009). Amygdala inhibitory circuits and the control of fear memory. Neuron.

[40] Huber D, Veinante P, Stoop R (2005). Vasopressin and oxytocin excite distinct neuronal populations in the central amygdala. Science.

[41] Cassell MD, Gray TS, Kiss JZ (1986). Neuronal architecture in the rat central nucleus of the amygdala: a cytological, hodological, and immunocytochemical study. J Comp Neurol.

[42] Hoffman GE, Lyo D (2002). Anatomical markers of activity in neuroendocrine systems: are we all ‘fosed out’?. J Neuroendocrinol.

[43] Welkenhuysen M, Gligorijevic I, Ameye L, Prodanov D, Van Huffel S, Nuttin B (2013). Neuronal activity in the bed nucleus of the stria terminalis in a rat model for obsessive-compulsive disorder. Behav Brain Res.

[44] Shekhar A, Truitt W, Rainnie D, Sajdyk T (2005). Role of stress, corticotrophin releasing factor (CRF) and amygdala plasticity in chronic anxiety. Stress.

[45] Regev L, Baram TZ (2014). Corticotropin releasing factor in neuroplasticity. Front Neuroendocrinol.

[46] Su J, Tanaka Y, Muratsubaki T, Kano M, Kanazawa M, Fukudo S (2015). Injection of corticotropin-releasing hormone into the amygdala aggravates visceral nociception and induces noradrenaline release in rats. Neurogastroent Motil.

[47] Hsu DT, Chen FL, Takahashi LK, Kalin NH (1998). Rapid stress-induced elevations in corticotropin-releasing hormone mRNA in rat central amygdala nucleus and hypothalamic paraventricular nucleus: an in situ hybridization analysis. Brain Res.

[48] Wang SS, Yan XB, Hofman MA, Swaab DF, Zhou JN (2010). Increased expression level of corticotropin-releasing hormone in the amygdala and in the hypothalamus in rats exposed to chronic unpredictable mild stress. Neurosci Bull.

[49] Helfferich F, Palkovits M (2003). Acute audiogenic stress-induced activation of CRH neurons in the hypothalamic paraventricular nucleus and catecholaminergic neurons in the medulla oblongata. Brain Res.

[50] Makino S, Shibasaki T, Yamauchi N, Nishioka T, Mimoto T, Wakabayashi I (1999). Psychological stress increased corticotropin-releasing hormone mRNA and content in the central nucleus of the amygdala but not in the hypothalamic paraventricular nucleus in the rat. Brain Res.

[51] Jones M, Dilley J, Drossman D, Crowell MD (2006). Brain–gut connections in functional GI disorders: anatomic and physiologic relationships. Neurogastroent Motil.

[52] Niedringhaus M, Jackson PG, Evans SR, Verbalis JG, Gillis RA, Sahibzada N (2008). Dorsal motor nucleus of the vagus: a site for evoking simultaneous changes in crural diaphragm activity, lower esophageal sphincter pressure, and fundus tone. Am J Physiol Regul Integr Comp Physiol.

